# Community detection in large scale congested urban road networks

**DOI:** 10.1371/journal.pone.0260201

**Published:** 2021-11-29

**Authors:** Seyed Arman Haghbayan, Nikolas Geroliminis, Meisam Akbarzadeh

**Affiliations:** 1 Department of Transportation Engineering, Isfahan University of Technology, Isfahan, Iran; 2 Ecole Polytechnique Federale de Lausanne (EPFL), School of Architecture, Civil and Environmental Engineering (ENAC), Urban Transport Systems Laboratory (LUTS), Lausanne, Switzerland; Monash University, AUSTRALIA

## Abstract

Traffic congestion in large urban networks may take different shapes and propagates non-uniformly variations from day to day. Given the fact that congestion on a road segment is spatially correlated to adjacent roads and propagates spatiotemporally with finite speed, it is essential to describe the main pockets of congestion in a city with a small number of clusters. For example, the perimeter control with macroscopic fundamental diagrams is one of the effective traffic management tools. Perimeter control adjusts the inflow to pre-specified regions of a city through signal timing on the border of a region in order to optimize the traffic condition within the region. The precision of macroscopic fundamental diagrams depends on the homogeneity of traffic condition on road segments of the region. Hence, previous studies have defined the boundaries of the region under perimeter control subjected to the regional homogeneity. In this study, a cost-effective method is proposed for the mentioned problem that simultaneously considers homogeneity, contiguity and compactness of clusters and has a shorter computational time. Since it is necessary to control the cost and complexity of perimeter control in terms of the number of traffic signals, sparse parts of the network could be potential candidates for boundaries. Therefore, a community detection method (Infomap) is initially adopted and then those clusters are improved by refining the communities in relation to roads with the highest heterogeneity. The proposed method is applied to Shenzhen, China and San Francisco, USA and the outcomes are compared to previous studies. The results of comparison reveal that the proposed method is as effective as the best previous methods in detecting homogenous communities, but it outperforms them in contiguity. It is worth noting that this is the first method that guarantees the connectedness of clusters, which is a prerequisite of perimeter control.

## Introduction

Since over a decade ago, Network or Macroscopic Fundamental Diagram (MFD) is recognized as a promising tool for monitoring vehicular traffic conditions and implementing control strategies with the goal of analyzing and alleviating congestion problems at network scale. An MFD relates the link-averaged traffic flow of a certain region of a city to its link-averaged traffic density. Parameters of an MFD include free flow speed, critical density, capacity and queue discharge rate which all pertain to a specific urban region. It has been shown that MFDs are concave and not highly sensitive to demand pattern [1]. Therefore, setting traffic density at an optimum value (i.e. critical density) would set the flow at its maximum which implies highest utilization of the capacity provided by the network. This control process is called perimeter control. Traffic density within a region may be controlled by either signal time setting or cordon pricing. In signal time setting, the amount of green time allocated to the lights on the borders of region under attention is et such that the value of inflow and outflow yield the desired value of vehicle accumulation within the region. In cordon pricing, number of vehicles entering the region is controlled via tolls drivers have to pay to be permitted to enter the region. These methods have been extensively explained in several studies [2–5].

Accurate estimation of MFD parameters is essential for efficient implementation of perimeter control. This estimation is carried out by fitting a predetermined function to average flow and density values; hence, it is evident that shape and amount of dispersion of MFD affect the accuracy of the estimation. The precision of macroscopic fundamental diagrams depends on the homogeneity of traffic condition on road segments of the region [6–8]. Accordingly, various studies have tried to detect sub-regions which yield best possible MFDs in terms of dispersion. Geroliminis and Sun investigated the variance of road density as a heterogeneity metric and obtained a well-defined MFD [9]. Ambühl et al. measured heterogeneity by proposing a functional form of MFD based on smooth approximation of uMFD (the analytical upper bound of macroscopic fundamental diagram). The smoothing parameter of a functional form reveals the degree of heterogeneity as a distance between MFD and its upper bound [10]. Besides their study, a novel technique called re-sampling method has been proposed, which is used when the shape of MFD is severely affected by heterogeneity due to insufficient input data [11]. Ji and Geroliminis [12]; Saeedmanesh and Geroliminis [13] have also addressed the network partitioning problem in order to reduce heterogeneity.

Ji and Geroliminis developed a method for partitioning URNs consisting three consecutive algorithms. To do so, they first provided an over-segmenting of the network by a Normalized cut algorithm. Secondly, a merging algorithm was developed based on initial segmenting to obtain a rough partitioning of the network. Finally, a boundary adjustment algorithm was designed to further improve the quality of partitioning by decreasing the variance of road density while maintaining the spatial compactness of clusters. They showed that their method had outperformed k-means clustering in a real URN case study [12]. In addition, Saeedmanesh and Geroliminis proposed a method for partitioning a URN into homogenous connected sub-regions based on traffic density in road segments. They first identified connected homogeneous areas around each road of the URN. Each sequence of roads, i.e. ‘snake’, was built by starting from a road and iteratively adding an adjacent road based on its resemblance to previously added roads in the sequence. Afterwards, based on the sequences obtained from the first step, a similarity measure was defined between each pair of the links in the network. The similarities were intended to put more weight on neighboring links and facilitate the connectivity of clusters. In the end, they utilized a symmetric non-negative matrix factorization framework to assign links to proper clusters with high intra-similarity and low inter-similarity [9]. Later, Saeedmanesh and Geroliminis developed the method to a dynamic case in order to incorporate delay propagation throughout the URN. Both attempts were successful in defining homogenous compact clusters for a real URN i.e. Shenzhen, China [13].

### Clusters

Communities (also known as clusters) of a network are subsets of nodes densely connected to each other and sparsely connected to other nodes of the network [14]. In the field of urban transportation networks, community detection has been employed for structural analysis [15], resilience and vulnerability analysis [16], perimeter control or route guidance [2, 17, and network design 18]. Besides, traffic congestion in urban road networks is still seen as a major problem imposing damaging effects on travel time, fuel consumption, safety and the environment. In general terms, spatial clustering is a well-studied problem in diverse fields of quantitative sciences. Depending on the nature of the problem and type of data, e.g. climate zoning [19], regionalization [20], geography [21], etc., different approaches including density-based [22], distance-based [23], and hierarchical clustering [24] have been proposed in the literature.

This paper primarily aims to find the sub-regions of urban road networks satisfying the following five criteria: (**a**) internal homogeneity in terms of traffic density, (**b**) external heterogeneity with other sub-regions, (**c**) sparse connection to their neighbor sub-regions, (**d**) connectedness (i.e. the trip length between any pair of nodes in a sub-region is a finite number), and (**e**) computational efficiency of the method, in which the shorter running time would offer an advantage in adaptation of the perimeter control boundary to the real-time traffic situation. This is regarded a challenging task, notwithstanding the heterogeneity caused by the classification of road segments and the spatial distribution of origins and destinations in the spatial distribution of congestion. To achieve these goals, the clusters detected by a well-established community detection method based on density discrepancy were modified. Thereafter, the method was applied to two previously studied URNs and the results were compared to those of existing methods with an emphasis on the advantages of our method. It is worth mentioning that criteria (c) and (d) have not been taken into account in previous studies.

## Methods

The importance of satisfying the above-mentioned criteria led us to propose an algorithm based on a community detection technique as described below. Algorithm 1 indicates the pseudocode of our proposed algorithm has three major steps (A, B and C): providing a weighted graph, implementing the community detection method (Infomap) and modifying detected communities to ensure minimum possible heterogeneity. Such steps are iterative because the community detection method (as explained in the following subsection) is unsupervised and when it was initially applied, numerous communities of various sizes were achieved. Therefore, coarse graining of the communities was continued and the algorithm was re-run until the desired number of communities was reached. It is worth noting that in this paper, the terms community and cluster are used interchangeably. We used Infomap because we found it suitable and also superior to other methods for our network. We found its suitability based on our five criteria mentioned in previous section. Due to its algorithm for formation of clusters (which we explain hereafter) Infomap guarantees the connectedness of the clusters. We added some steps assure the sparseness of the borders and enhance the homogeneity of each cluster. Superiority of Infomap with regard to other well-known clustering methods applicable to urban road networks is already established in [16] and [25].

Algorithm 1. Pseudocode of the proposed algorithm.

Algorithm

*Input*:

Graph *D*_*z*_ = (*V*, *E*) where *V* is a set of nodes and *E* is a set of links in the dual representation of the URN

*C* ← *V* (Assuming each node as a cluster prior to the 1^st^ iteration) *v* ϵ *V*

*Z* = 1

*While size(C)* > 1 *do*:

 A. Setting the weight of links in graph *D*_*z*_

  *For each i* ϵ *C do*:

   *S* ← Set of nodes neighboring *i*

   *For j ϵ S do*:

    $w_{ij}^{D_{z}}={\left[{\bar{k}}_{i}^{c}-{\bar{k}}_{j}^{c}\right]}^{-\gamma }$

 B. Applying Infomap to the weighted graph *D*_*z*_

  *C*_*Infomap*_ = {*c*_*1*_, *c*_*2*_, *…*, *c*_*m*_} *c*_*m*_ ⊂ *V* (Set of clusters identified by Infomap)

 *If z >1 then*:

  C. Modifying clusters to reduce the variance

   *C*_*modified clusters*_: Ø (Make the set empty for the modified clusters)

   *For c*_*m*_ ϵ *C*_*Infomap*_ *do*:

    *R = {v|v ϵ c*_*m*_*}* (Set of nodes that belong to the cluster *c*_*m*_ (labelled *c*_*m*_))

    *N* = *n*(*R*) (The number of elements in the set *R*)

    *MV = 0*

    *While N ≥ 1 do*:

     *$R_{N}^{\prime }=\{r\subset R|n(r)=N\}$* (Generate all subsets of size *N* from the set *R*)

     *For $r\in R_{N}^{\prime }$ do*:

      *If* the subgraph of nodes *r* is connected *then*:

       $c_{m}^{\prime }=\{v|v\epsilon r\}$ (Assign label $c_{m}^{\prime }$ to nodes *r* (as a subset of cluster *c*_*m*_)) $c_{m}^{\prime }\subset c_{m}$

       $N_{c_{m}^{\prime }}=n(c_{m}^{\prime })$ (The number of elements in set $c_{m}^{\prime }$)

       $MV_{c_{m}^{\prime }}=N_{c_{m}^{\prime }}{\left[var(c_{m})-var(c_{m}^{\prime })\right]}^{2}$

       *If $MV_{c_{m}^{\prime }}>MV$ then*:

        *R*_*best*_ = *r*

        $MV=MV_{c_{m}^{\prime }}$

     *N = N– 1*

    *c*_*m*_ = {*v*|*v ϵ R*_*best*_} (Assign label *c*_*m*_ to nodes *R*_*best*_)

    Add *c*_*m*_ to *C*_*modified clusters*_

    *for v* ϵ (*R* − *R*_*best*_) *do*:

     Assigning a new label to node *v* (Assume node *v* as a separate cluster)

     Adding *v* with its new label to *C*_*modified clusters*_

    *C ← C*_*modified clusters*_

     *Else*:

    *C ← C*_*Infomap*_

   Updating graph *D*_*Z*_ based on set *C* (the nodes with same label are merged together)

  *Z = Z +1*

**Fig 1:**
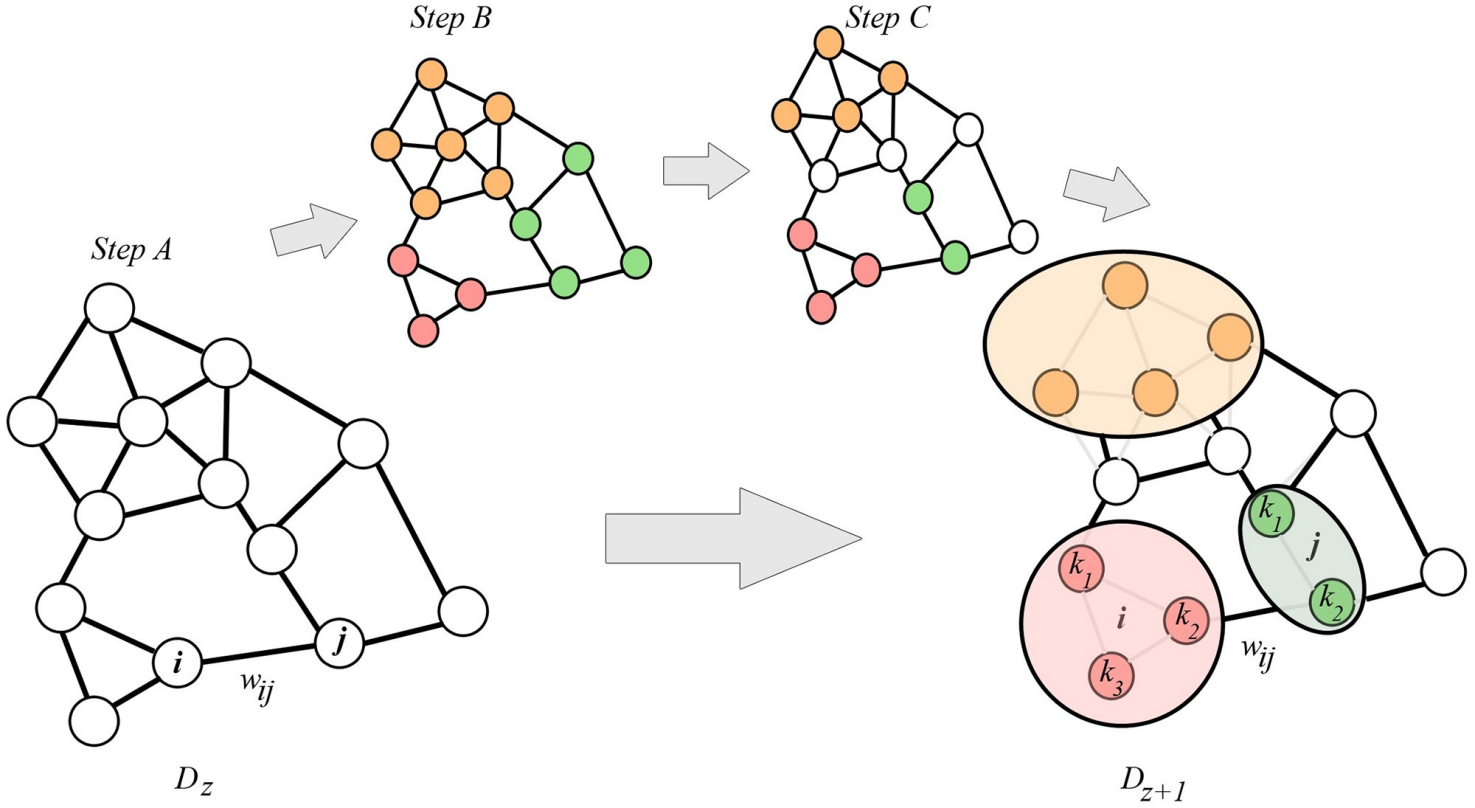
The agglomeration process in three steps.

As depicted in Algorithm 1, prior to algorithm initiation, the URN was transformed into a graph using a dual approach (*D*_*z*_) in which each road segment was a node and intersections were links [26]. Moreover, an agglomerative approach was adopted in which each node was initially assumed to be a separate community and then for every iteration *z*, similar nodes were agglomerated into the same communities. Fig 1 depicts the procedure of agglomeration in a graph whose nodes were agglomerated by those three steps. However, as shown in this figure, each white node is considered as a different community and nodes collected into a similar community are represented by the same color.

In the first step, a graph was made by the dual approach (1^st^ iteration) or obtained from a previous iteration. Also, it was found that its nodes were either road segments (1^st^ iteration) or clusters, each consisting some road segments. Therefore, in order to incorporate the density discrepancy of neighbor nodes in the community detection method in the next step, the weights of links were set as follows:

$$w_{ij}^{D_{z}}={\left[{\bar{k}}_{i}^{c}-{\bar{k}}_{j}^{c}\right]}^{-\gamma }$$ (1)

Where $w_{ij}^{D_{z}}$ is the weight of the link connecting node *i* to *j* in graph *D*_*z*_. ${\bar{k}}_{i}^{c}$ denotes the mean road density within the cluster (node) *i*. Accordingly, in the first iteration, each road was assumed to be a separate cluster. Finally, *γ* is a tuning parameter reflecting the importance of density discrepancy in setting the communities.

By definition, the community detection methods consider the intensity of connectivity of nodes in discovering communities. However, in weighted graphs, the weight of links is also considered so that a pair of nodes connected through a higher weight link are assumed to be “more connected” than a pair connected by a lower weight link. Thus, in this case, we lead the community detection method into setting road segments of similar density values in the same clusters.

### Community detection method

At this stage, the weighted graph *D*_*z*_ is developed to apply the community detection method (Step B). For this purpose, the Infomap was selected as its computational performance and accuracy is superior to many other methods [16, 25, 27, 28]. The Infomap minimizes the descriptive length required for enlisting the path traversed throughout the network by a random walker [29]. Intuitively, a random walker takes more steps in the parts of the network that are more connected. Alternatively, once inside a cluster, the random walker is more likely to take its next step within rather than outside the cluster. Hence, running the random walk several times and tracking the walker would reveal the clusters of the network.

The lower bound of the average descriptive length is calculated based on the “map equation” depicted in Eq 2. The map equation states that the average descriptive length of walks under the cluster configuration M (*L(M)*) is equal to the sum of the average number of bits required to describe the movements of a random walker between clusters (denoted by ↷ subscript) and the average number of bits required to describe their inter-cluster movements (denoted by ↻ subscript).


$$L\left(M\right)=q_{↷}H\left(Q\right)+\sum_{c=1}^{m}P_{↻}^{c}H\left(P^{c}\right)$$ (2)

The first term on the right-hand side of the map equation describes the average number of bits required for describing the inter-cluster steps. *q*_↷_ denotes the probability of switching clusters in each step, which is equal to the sum of probabilities of the random walker exiting cluster *c* (*q*_*c*↷_). On the other hand, the average length of a code word required for describing the states of a random variable *X* occurring with probability *q*_*c*_ is at least equal to the entropy of *X* i.e. *H*(*X*) [30]. Therefore, the entropy of movements among clusters could be obtained from Eq 3.


$$H\left(Q\right)=\sum_{c=1}^{m}\frac{q_{c↷}}{q_{↷}}\text{log}\left(\frac{q_{c↷}}{q_{↷}}\right)$$ (3)

The second term on the right-hand side of the map equation shows the average number of bits required to describe inter-cluster movements, which is equal to the entropy of inter-cluster movements. H(P^c^) is the entropy of intra- cluster movements c. ${\text{P}}_{↻}^{\text{c}}$ is the fraction of intra- cluster movements and the possibility of exiting the cluster c, which could be computed from Eq 4.


$$P_{↻}^{c}=q_{c↷}+\sum_{j\in c}p_{j}$$ (4)

In other words, $P_{↻}^{c}$ is the amount of time a random walker spends in a cluster before existing it *c*. *p*_*j*_ is the probability of the visiting node t, which is equal to the sum of visit rates on links (*q*_*ij*_) over all source nodes *i*:

$$p_{j}\;=\sum_{i}q_{ij}\;=\sum_{i}p_{i}\cdot p_{ij}$$ (5)

*p*_*ij*_ denotes the conditional probability that the random walker moves from node i to node j. This is where the link weights come into equation.


$$p_{ij}=\frac{w_{ij}}{\sum_{j}w_{ij}}$$ (6)

As URNs are directed, the random walker might get stuck in a dangling node, i.e. a node with only incoming links. To avoid this situation and ensure the steady-state distribution, teleportation is introduced to the random walk. Wirth the introduction of teleportation, the random walker is converted into a random surfer: at each time step with probability 1 –*τ*, the random surfer follows one of the outgoing links from node s to its adjacent node t with a probability corresponding to the weights of the outgoing link connecting *i* to *j* (*w*_*ij*_). With the probability *τ*, the random surfer teleports to a random node with uniform probability anywhere in the network. If node *s* has no outgoing links, the surfer would teleport with probability 1 [31]. Therefore, the probability that the random surfer reaches node *j* (*p*_*j*_) is calculated as follows:

$$p_{j}=\left(1-\tau \right)\sum_{j}p_{i}\cdot p_{ij}\;+\;\tau \;\frac{\sum_{i}w_{ij}}{\sum_{i.j}w_{ij}}$$ (7)

This is the mechanism by which the choice of weights has a bearing on the number and structure of clusters. *H*(*P*^*c*^) in Eq 2 is the entropy of internal movements in clusters, which is calculated as follows:

$$H\left(P^{c}\right)=\frac{q_{c↷}}{q_{c↷}+\sum_{j\in c}p_{j}}\text{log}\left(\frac{q_{c↷}}{q_{c↷}+\sum_{j\in c}p_{j}}\right)+\sum_{i\in c}\frac{p_{i}}{q_{c↷}+\sum_{j\in c}p_{j}}\text{log}\left(\frac{p_{i}}{q_{c↷}+\sum_{j\in c}p_{j}}\right)$$ (8)

By combining all these values in the map equation, the average description length for one step could be obtained under a specific cluster configuration *M*.

### Homogeneity measures

Similar to previous studies, we utilized *TV*_*N*_ to evaluate the performance of clustering and comparing methods. According to Eq 10, *TV*_*N*_ indicates the ability of a clustering setting to partition the URN into a homogeneous sub-region.


$$TV_{N}=\frac{TV_{partitioned}}{TV_{unpartitioned}}=\frac{\sum_{m=1}^{N_{c}}N_{c_{m}}\times var\left(c_{m}\right)}{N\times var\left(c\right)}$$ (9)

In this case, *N* is the number of nodes in graph *D*_*z*_, $N_{c_{m}}$ is the number of nodes in community *c*_*m*_ and *N*_*c*_ is the total number of communities in the setting under evaluation. *var*(*c*_*m*_) is the variance of road density within community *c*_*m*_ and *var*(*c*) is the variance of total road density (without partitioning).

The clusters detected by the Infomap are modified in order to assure minimum possible heterogeneity among all road segments of a community (step C). As shown in Algorithm 1, this goal was fulfilled by finding the best subset of a cluster maximizing the $MV_{c_{m}^{\prime }}$, as defined in Eq 11. This equation reveals improvement in variance of road density in the case that cluster *c*_*m*_ only consists of the nodes in subset $c_{m}^{\prime }$. The size of the subset ($N_{c_{m}^{\prime }}$) was taken into account in order to prevent communities from complete decomposition and to highlight subsets with further nodes. It is noteworthy that the subsets of a cluster were found by generating all subsets of a set that contained nodes belonged to a cluster (with the same label) and checking their contiguity in a subgraph in which there were no other nodes of cluster. This means that a subset of cluster *c*_*m*_ ($c_{m}^{\prime }$) was defined as a subset containing connected nodes with the label *c*_*m*_.


$$MV_{c_{m}^{\prime }}=N_{c_{m}^{\prime }}{\left[var\left(c_{m}\right)-var\left(c_{m}^{\prime }\right)\right]}^{2}$$ (10)

In the next step, the nodes of the best subset (maximum amount of $MN_{c_{m}^{\prime }}$) were merged and held as a cluster while another nodes remained unchanged as separate clusters.

### Case of incomplete information

It is not economically feasible to install traffic detectors in all urban roads. Hence, the traffic data (speed or density) of some roads would be unavailable when it comes to network analysis and community detection. In fact, the missing data induce uncertainty about the weight links of graph *D*_*z*_ (step A) and consequently prevent the random walker from moving based on density discrepancy (step B). Therefore, the random walk was limited to moves in the roads for which data was available. It was conducted by providing new connections in a specified maximum distance between non- neighbor roads and roads where the random walker had to pass a missing node. This distance precludes generating disjointed parts in the path of a random walker for low data penetration rate. Also, in order to guarantee the contiguity of communities, a penalty was set for the random walker’s movement through these connections. Therefore, the weight links of the graph *D*_*z*_ for incomplete cases would be obtained as follows:

$$w_{ij}^{D_{z}}={\left[\delta^{d_{ij}-1}\times \left({\bar{k}}_{i}^{c}-{\bar{k}}_{j}^{c}\right)\right]}^{-\gamma }$$ (11)

Where, *δ* is the penalty value and *d*_*ij*_ is the shortest path between nodes *i* and *j*. It should be noted that the graphs of incomplete cases only contained nodes for which data was available. Thus, for each iteration, the shortest path between nodes was independently calculated from another graph that had all nodes (even the missing nodes) and its weight links were 1 ($w_{ij}^{D_{z}}=1$).

## Results

The proposed method was applied to the network of San Francisco, USA and Shenzhen, China. These networks were used to test the previous methods of community detection for the application of perimeter control based on MFD. Data on San Francisco was derived from a simulation and the data on Shenzhen was gathered from a database of 20000 taxi trajectories. In this section, first the results are explained and compared to Infomap in order to show the effectiveness of the modification step. Then, a comparison is drawn between the present findings and those of previous methods.

Table 1 shows the effect of *γ* on homogeneity, highlighting the fact that a larger number of clusters would improve homogeneity in the values of *TV*_*N*_. In fact, given the interaction effect of intensity of connectivity and density discrepancy on random walker movement, different values of *γ* in networks were tested with various structures. Therefore, the optimum clustering was considered for the case where more homogeneity was achieved with a lower number of clusters. As can be seen, the optimum values of *γ* were 3 and 2 for San Francisco and Shenzhen, respectively. It is worth noting that values greater than 4 could not be used because clusters emerged in a road segment.

**Table 1:** Clustering results.

	San Francisco		Shenzhen	
*γ*	#clusters	*TV*_*N*_	#clusters	*TV*_*N*_
1	5	0.37	5	0.69
	3	0.36	4	0.71
2	6	0.19	6	0.42
	4	0.21	3	0.54
3	5	0.17	6	0.45
	2	0.20	3	0.53
4	4	0.21	6	0.44
	3	0.23	4	0.57

**Fig 2:**
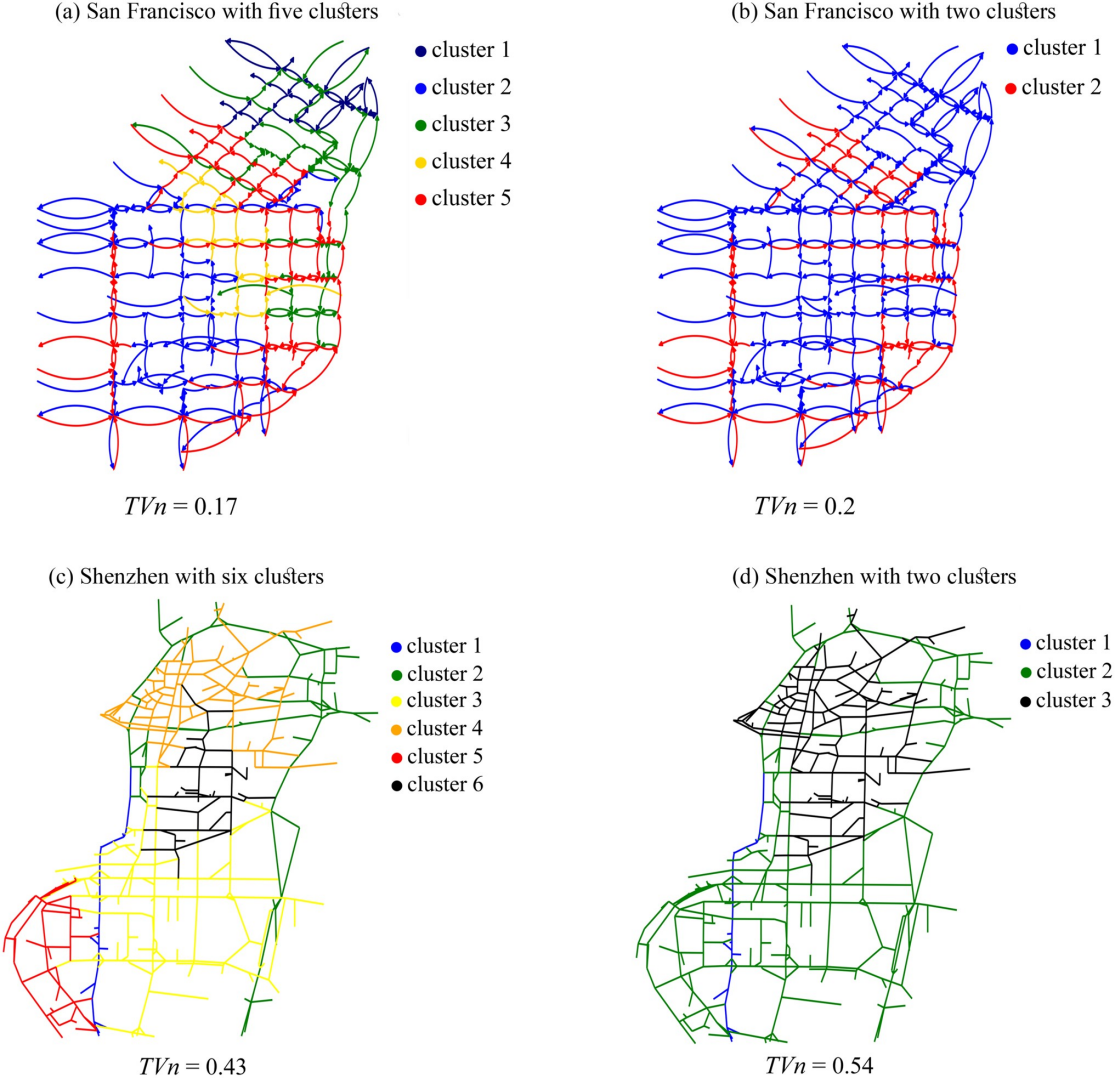
Clustering results for San Francisco and Shenzhen.

Fig 2 depicts clusters in both studied cities. The contiguity of communities is evident in this figure. Figures on the right show a higher number of clusters than those on the left. A higher number of clusters improves the homogeneity of clusters but the clusters may be too small for a perimeter control. This situation is shown in cluster 1 of San Francisco and Shenzhen in (Fig 2a and 2c), respectively. However, the minimum value of *TV*_*N*_ obtained with a reasonable number of clusters is of theoretical value in assessing the quality of a method and comparing their ability in detection of homogeneous communities.

### Case of incomplete information

**Fig 3:**
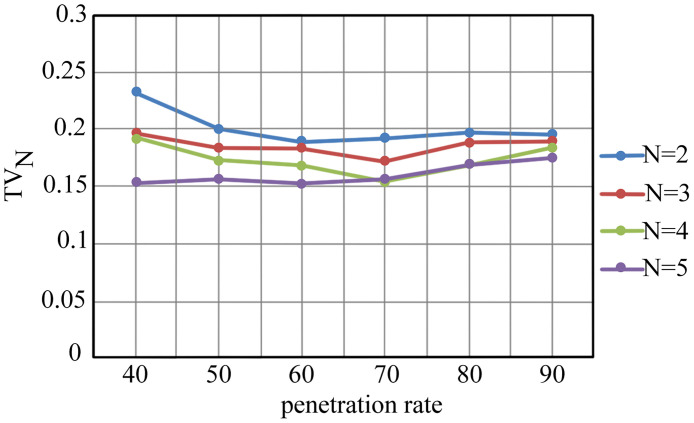
The effect of incomplete information on effectiveness of the method (*δ* = 3).

The functionality of the proposed method was scrutinized at different levels of data availability. Fig 3 illustrates *TV*_*N*_ variation as the penetration rate increases from 40 to 90%. It is clear that the proposed method is robust even for incomplete information as the homogeneity index of clusters does not change significantly due to variations in the penetration rate. The maximum connection distance is 3.

### Dynamic clustering

**Fig 4:**
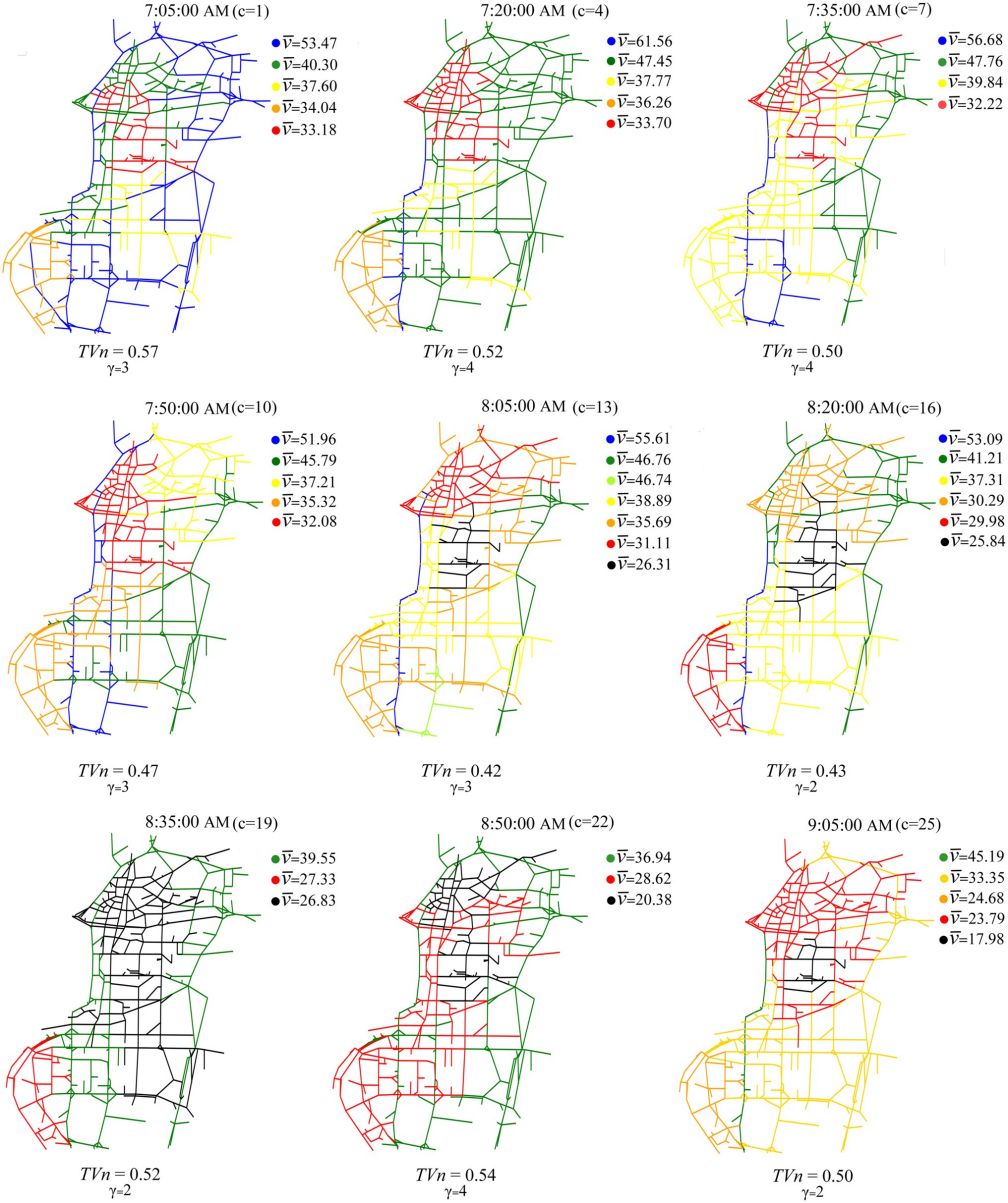
Dynamic clustering of Shenzhen network using the proposed method.

Data gathered in Shenzhen included traffic information aggregated and averaged over 5-min time intervals. This data was used for dynamic clustering of the network. Fig 4 shows the results for 15 min time intervals. $\bar{v}$ [km/hr] shows the average speed in each cluster, which is an indicator of cluster discrepancy in terms of their traffic condition.

### Comparison with Infomap

**Fig 5:**
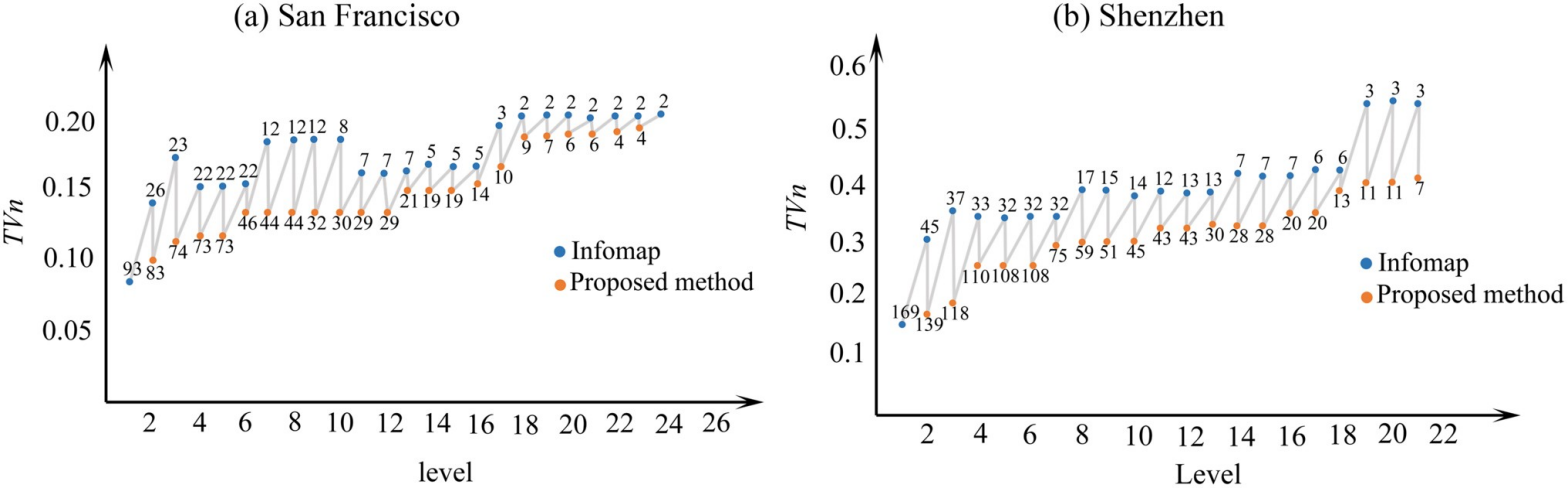
Homogeneity of clusters under Infomap and the proposed method.

The benefits of modification made to the Infomap become evident by comparing the homogeneity of clusters in each method. Fig 5 shows *TV*_*N*_ values derived from the original Infomap and our method. Horizontal axis shows the number of iterations (5 for San Francisco and 22 for Shenzhen).

Numbers beside the circles indicate the number of clusters in that iteration. As shown in Fig 5, Infomap modifications improves the homogeneity of clusters.

### Comparison with previous studies

Table 2 shows *TV*_*N*_ values obtained from previous methods. A comparison of values listed in this table and Table 1 suggests 10% difference between the homogeneity of clusters detected in proposed method and the one introduced by Saeedmanesh and Geroliminis. However, unlike previous methods, the presented method guarantees the contiguity of clusters, which is crucial for implementing the perimeter control. Therefore, it was possible to find connected clusters that are almost as homogeneous as clusters identified by previous methods.

**Table 2:** *TV*_*N*_ results of previous methods.

	San Francisco		Shenzhen	
	#clusters	*TV*_*N*_	#clusters	*TV*_*N*_
Ji and Geroliminis (2012)	3	0.85	4	0.89
Saeedmanesh and Geroliminis (2016)	2	0.17	2	0.74
	3	0.17	3	0.60
	4	0.17	4	0.57
	5	0.16	5	0.45
Saeedmanesh and Geroliminis (2017)	2	0.17	2	0.48
	3	0.16	3	0.38
	4	0.15	4	0.37

## Conclusion

In this study, a new method for clustering real world urban road networks was proposed. The proposed method is based upon well-established Infomap but enforces modifications which apparently enhance the quality of results. The main application of this method is dividing urban areas into homogenous and connected regions. This would in turn enable urban traffic managers to implement perimeter control more accurately and effectively. Using macroscopic fundamental diagrams, perimeter control sets the inflow of an urban region to maximize the spatial average of traffic flow in the region. Maximizing the spatial average of traffic flow indicates increasing the utilization of provided capacity through road infrastructure.

The method proposed in this paper has several advantages over existing methods. For instance, while its regions are as homogenous as those achieved by the best previous methods, it guarantees connectedness and computational efficiency. The proposed method was tested under incomplete information in which traffic data is available for only a fraction of links of a network. It was shown that the proposed method is fairly robust versus lack of input data.
